# Zeolite-iron oxide integrated interdigitated electrode sensor for diagnosing cervical cancer

**DOI:** 10.1016/j.heliyon.2024.e31851

**Published:** 2024-05-23

**Authors:** Ling Li, Subash C.B. Gopinath, Thangavel Lakshmipriya, Sreeramanan Subramaniam, Periasamy Anbu

**Affiliations:** a Obstetrics and Gynecology, Xi'an Forth Hospital, Xi'an, 710004, China; b Faculty of Chemical Engineering & Technology, Universiti Malaysia Perlis (UniMAP), 02600, Arau, Perlis, Malaysia; c Institute of Nano Electronic Engineering, Universiti Malaysia Perlis (UniMAP), 01000, Kangar, Perlis, Malaysia; d Micro System Technology, Centre of Excellence (CoE), Universiti Malaysia Perlis (UniMAP), Pauh Campus, 02600, Arau, Perlis, Malaysia; e Centre for Chemical Biology, Universiti Sains Malaysia, Bayan Lepas, 11900, Penang, Malaysia; f Center for Global Health Research, Saveetha Medical College & Hospital, Saveetha Institute of Medical and Technical Sciences (SIMATS), Thandalam, Chennai, 602 105, Tamil Nadu, India; g School of Biological Sciences, Universiti Sains Malaysia, Georgetown, 11800, Penang, Malaysia; h Department of Biology, Faculty of Science and Technology, Universitas Airlangga, Surabaya, 60115, Indonesia

**Keywords:** Cervical cancer, Nanomaterial, DNA sensor, Interdigitated electrode

## Abstract

Cervical cancer is caused by changes in the cervix that lead to precancerous cells and eventually progress to cancer. Human papillomavirus (HPV) infections are the primary cause of cervical cancer. Early detection of HPV is crucial in preventing cervical cancer, and regular screening for HPV infection can identify cell changes before they develop into cancer. While Pap smear tests are reliable for cervical cancer screening, they are critical, expensive, and labor-intensive. Therefore, researchers are focusing on identifying blood-based biomarkers using biosensors for cervical cancer screening. HPV strains 16, 45, and 18 are common culprits in cervical cancer. This study aimed to develop an HPV-16 DNA biosensor on a zeolite-iron oxide (zeolite-IO) modified interdigitated electrode (IDE) sensor. The DNA probe was immobilized on the IDE through amine-modified zeolite-IO, enhancing the hybridization of the target and DNA probe. The detection limit of the DNA-DNA duplex was found to be 7.5 pM with an R^2^ value of 0.9868. Additionally, control experiments with single and triple mismatched sequences showed no increase in current responses, and the identification of target DNA in a serum-spiked sample indicated specific and selective target identification.

## 1 Introduction

Cervical cancer originates from the cells of the cervix and can spread to nearby areas such as the rectum, bladder, vagina, uterus, and pelvic lymph nodes [1,2]. Typically, cervical cancer develops gradually; initially, cervical cells undergo abnormal changes that progress to cancerous cells [3]. However, not all abnormal cells progress to cancer, underscoring the importance of identifying and treating problematic cells before they become cancerous. Human papillomavirus (HPV) infection is the primary cause of cervical cancer, transmitted through sexual contact via oral, vaginal, or anal routes. HPV prevalence is increasing worldwide, particularly among women under 25 in countries such as India, Latin America, Mongolia, and China [[4], [5], [6], [7]]. Numerous HPV types have been identified, with types 70, 68, 66, 59, 58, 56, 52, 51, 45, 39, 35, 34, 33, 31, 18, and 16 considered highly risky. Notably, HPV 16 and 18 are responsible for 70 % of cervical cancer cases, highlighting the importance of early detection to prevent cervical cancer [8,9]. Regular screening helps identify cell changes before they become cancerous, particularly detecting precancerous lesions early to prevent cancer progression. Although cytologic screening of cervical cells is commonly used for this purpose, it may not always provide distinct results for affected cells. In such cases, a biopsy and colposcopy are recommended for further confirmation, despite their complex procedures and costs. Therefore, a highly sensitive biosensing system is essential for detecting HPV DNA, capable of distinguishing between low and high-risk HPV types, thereby enhancing treatment, triage, and follow-up for affected patients [3,10]. DNA-based sensors have shown greater interest recently as it is the detection test for genomic identification and as early identification for various diseases including cancer [[11], [12], [13], [14], [15]]. The detection was identified by changes that happened during the hybridization between the target solution and the DNA-functionalized electrode. DNA-based sensors use the nucleic acid amplification method to increase the biosensor responses [[16], [17], [18]]. DNA template biosensors can decorate with the DNA formulation like DNA origami, and DNA tetrahedron is to identify the DNA molecule [19,20]. Many DNA-based sensors are effectively applied in various electrochemical techniques such as impedance spectroscopy, chronoamperometry, and capacitance [[21], [22], [23]]. These electrochemical techniques have a greater ability to provide the label-free, portable, and real-time measurement of DNA sensors. This research was focused on detecting HPV-16 specific DNA on Si–Al–Fe iron-modified interdigitated electrode (IDE) sensor.

The incorporation of nanomaterials provides a greater impact on disease identification in biosensing technology. Functionalized nanomaterials with unique chemical and physical properties have been developed as new biosensing techniques for biomedical applications [[24], [25], [26]]. Various hybrid and inorganic nanomaterials have become the basis for the high performance of biosensors with improved sensitivity, and selectivity. These advanced nanomaterials such as carbon nanotubes, silica gold, quantum dot, rare earth nanoparticles, iron, zeolite, and graphene are effectively applied in various biosensors for pH sensing, DNA, protein, bacteria, and virus identification [[27], [28], [29]]. Nanomaterials with electrochemical sensors, colorimetric sensors, surface plasmon resonance, and RAMAN spectroscopy help to identify various diseases for diagnosing cancer, and viral and bacterial infection [30]. In this research, zeolite-iron oxide (zeolite-IO) incorporated nanomaterial was utilized to attach HPV-16 DNA probe on IDE, which improves DNA immobilization and enhances the sensitivity of target DNA detection. Silicon (Si) is one of the established materials in biosensor development, silicon nanohybrids, and nanostructures have been extensively created for developing various biosensing applications [31]. Different dimensions of Si nanomaterials such as one-dimensional silicon nanowires, and zero-dimensional fluorescent Si nanoparticles, have been immobilized on various biosensors and helps to detect various targets [32,33]. Similarly, aluminum is an unavoidable material in biosensor development and in most cases, Al was used to fabricate the sensing surface due to its higher electrical conductivity. Iron-based nanomaterial such as iron nanoparticles is also used for surface functionalization of biomolecules on the sensing surface [34,35]. Due to their excellent biocompatibility, and low toxicity, iron nanomaterials are generally used for drug delivery cancer therapy, and cancer imaging. Alongside, it was found that a greater application of various biosensors such as Bisphenol A, Gas, and glucose sensors [35]. Here we incorporated three nanomaterials Si, Al, and Fe, which are extracted from the coal mine fly ash for developing HPV-16 DNA biosensors.

## 2 Materials and methods

### 2.1 Chemicals and biomolecules

(3-Aminopropyl)triethoxysilane (APTES), Sodium hydroxide (NaOH), and human serum Sulfuric acid (H_2_SO_4_) were received from Sigma Aldrich, USA. Whatmann filter paper, Ethanol was availed from Thermo Fisher Scientific, USA. Measurements of current volt for the interactions were recorded by Keithley 6487 Picoammeter. The following DNA probe and target were used to identify the HPV infection and synthesized from the local supplier. DNA probe: 5′-COOH(CH2)_6_GTCATTATGTGCTGCCATATCTACTTCAGA-3′; Target DNA: 5′-TCTGAAGTAGATATGGCAGCACATAATGAC-3′; Single mismatch: 5′-TCTGAAATAGATATGGCAGCACATAATGAC-3′; Triple mismatch: 5′-TCTGAAAGGGATATGGCAGCACATAATGAC-3′ [36].

### 2.2 Extraction of iron solution from coal fly ash

To remove the iron, 100 g of raw fly ash was dissolved in 1 L of water and stirred using a magnetic stirrer. The iron stuck on the rod was collected and repeated this process until all the irons are recovered. The collected irons were rinsed with water and added 25 % of sulfuric acid (H_2_SO_4_) and stirred continuously for 3 h at 100 °C. Whatmann filter paper was used to filter the iron solution.

### 2.3 Recovering sodium aluminosilicate from coal mine ash

To extract the sodium aluminosilicate,100 g of fly ash (iron removed) was added in 3 M of sodium hydroxide (NaOH, 1 L) and stirred for 4 h at 60 °C. After that, the sodium aluminosilicate was filtered by using Whatmann filter paper and used to synthesize zeolite-iron nanocomposite.

### 2.4 Synthesize of zeolite-IO by sol-gel process

Sol-gel process was used to synthesize zeolite-IO nanoparticle. Briefly, 500 mL of solidum aluminosilicate (pH 12) was added to a 1 L beaker and placed on the stirring plate (60 °C). Further, the extracted iron solution (pH 1) was added drop-by-drop to the sodium aluminosilicate until formation of the gel. At this point, the solution reached the neutral pH, and the clear formation was gel. The gel was further stirred overnight to get the uniform-sized particle. The next day gel was collected by using centrifugation at the speed of 15000 rpm for 15 min. Further, washed the gel ethanol and water and dried at 80 °C. The dried nanomaterial was analyzed under FESEM and FETEM observations to identify the size and composition of the material.

### 2.5 Optimization of HPV-DNA probe on zeolite-IO nanomaterial-modified IDE

HPV-DNA probe was attached to the electrode through the zeolite-IO nanomaterial. The following steps were followed for this immobilization process. (i) IDE was treated with potassium hydroxide (1 %) for 20 min and washed with distilled water; (ii) APTES-modified zeolite-IO (1 mg/mL) was placed on IDE for 2 h and then washout the excess APTES by ethanol; (iii) Different concentrations of COOH-ended DNA probe (200, 400, 600, 800, and 1000 nM) were immobilized; (iv) PEG-COOH (1 mg/mL) was added as a blocking material. In between each step, the surface was washed with buffer and the current-volt measurements were monitored.

### 2.6 Identification of HPV-DNA

To identify the HPV-target DNA, Different target DNA concentrations from 7.5 to 500 pM were dropped on HPV-DNA probe modified IDE. Then, the current-volt measurements were recorded after washing the surface with PBS. The current difference before and after hybridization was calculated to identify the limit of DNA detection.

### 2.7 Specific and selective identification of HPV-DNA

Specific DNA was identified by experimenting with single and triple mismatched, and complementary sequences. For that, instead of target DNA, single- and triple-mismatched and complete sequences were independently placed on HPV-DNA probe modified IDE. The surface was washed with PBS, and then the current-volt measurement was recorded.

## 3 Results and discussion

**Fig. 1:**
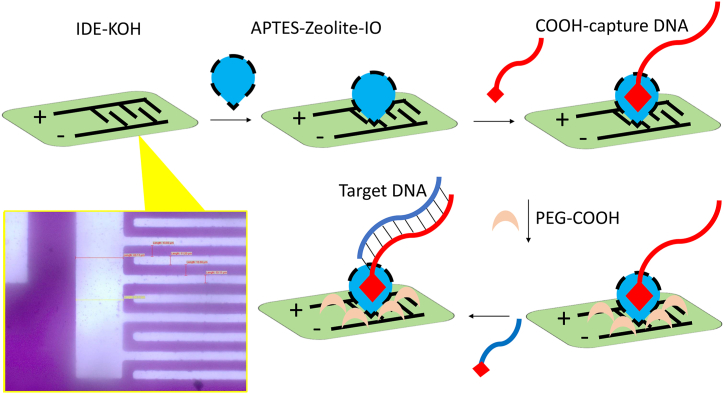
Schematic representation for surface functionalization of HPV-16 DNA biosensor. Interdigitated electrode was treated with KOH, and then amine-modified zeolite-IO was introduced. Further, COOH-ended DNA probe was added, and then the uncovered surfaces were blocked by PEG-COOH. Finally, target DNA was allowed to hybridize the DNA probe. The observed interdigitated electrode image under high-performance image is shown.

Early identification of cervical cancer with suitable biomarkers help to provide a better treatment and improve the patient's life. HPV-16 plays a major role in causing cervical cancer, and this research work developed a highly sensitive HPV-16 DNA biosensor on zeolite-iron oxide (zeolite-IO) modified interdigitated electrode sensor (IDE). Fig. 1 shows the schematic representation of the surface functionalization of the HPV-16 DNA biosensor. Initially, the surface of IDE was treated with potassium hydroxide (KOH) for efficient attachment of APTES. Research proved that uniform modification of APTES can be achieved on the KOH-treated sensing electrode. After KOH treatment, the surface was modified into amine-modified zeolite-IO through the link between KOH and APTES. On these zeolite–IO–modified surfaces, COOH-ended DNA probe was introduced, and then the electrodes were covered with PEG-COOH. In general, capture molecule immobilization plays a major part in enhancing the sensitivity and selectivity of biomolecular interaction. Higher attachment of the capture molecule on the sensing electrode enhances the biosensing performances. Here, amine-modified zeolite-IO was used for surface functionalization on the IDE, which increases the number of captured DNA on IDE. After these attachments, PEG-COOH was added to suppress the biofouling. In general amine-modified surfaces attract other biomolecules nonspecifically, and it is necessary to cover the excess APTES. PEG-based molecule reduces the nonspecific binding and also enhances the specific interaction of biomolecules. PEG-COOH binds the excess amine surfaces and reduces the biofouling of target molecules and helps for specific identification of target HPV-16 DNA.

### 3.1 Imaging of zeolite-IO by FESEM and FETEM

**Fig. 2:**
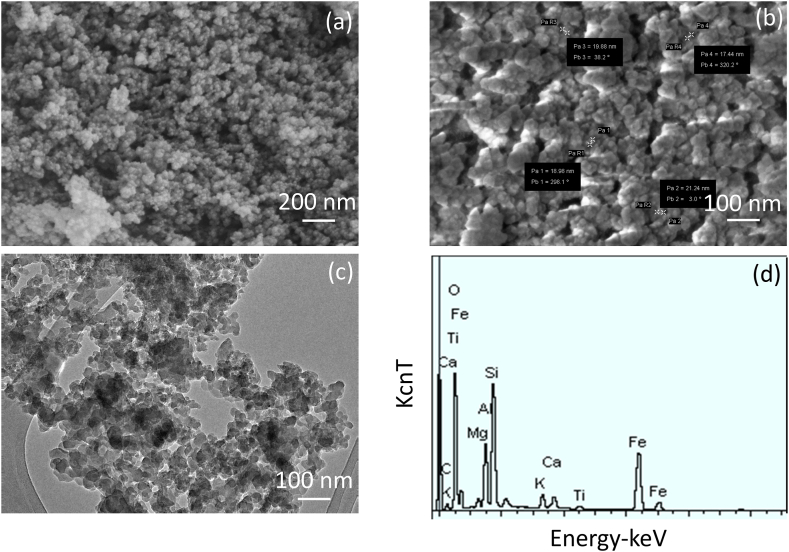
Imaging of extracted zeolite-IO. FESEM images at the magnifications of 200 nm (a) and 100 nm (b); (c) FETEM image at a magnification of 100 nm. The size of the nanoparticle is ∼20 nm with uniform distribution. (d) EDX analysis of zeolite-IO. The presence of major elements, Aluminum, silica, iron, and oxygen are in the extracted nanoparticle confirming the presence of zeolite-IO.

Imaging of extracted zeolite-IO was analyzed by FESEM and FETEM. Fig. 2a and b shows the FESEM images of zeolite-IO at the magnifications of 200 and 100 nm. The nanoparticles are approximately with the size of 20 nm, and they were distributed uniformly with a spherical shape. The FETEM image also confirms the size of the nanoparticle is ∼20 nm with a uniform distribution (Fig. 2c). Furthermore, EDX analysis was conducted to identify the presence of zeolite and iron in the nanoparticle. As shown in Fig. 2d, the major elements of Aluminum, silica, iron, and oxygen are in the extracted nanoparticle, and some traces amount of C and K are also in the nanoparticle. This result confirms the extraction of zeolite-IO from the coal fly ash.

### 3.2 DNA probe immobilization on zeolite–IO–modified IDE

**Fig. 3:**
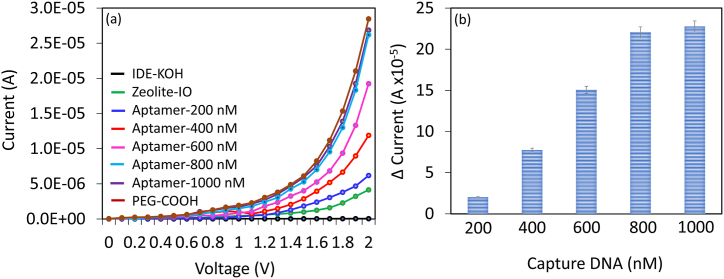
(a) DNA probe attachment on interdigitated electrode. (a) Current-volt measurement of different concentrations of DNA probe attachment on interdigitated electrode. With increasing the DNA probe, the current levels increased. (b) Difference in current level for DNA probe attachment. DNA probe with 800 and 600 nM shows about the similar current confirming the saturation of DNA probe immobilization on interdigitated electrode. Independent experiments were performed and averaged.

DNA probe Immobilization plays a major role in developing a highly sensitive biosensor. Here Zeolite-IO was used as the linker to immobilize the DNA probe on IDE. Fig. 3 shows the surface functionalization of HPV-16 DNA probe on zeolite-IO modified IDE. On these zeolite–IO–modified surfaces, different concentrations of DNA probe were tested and optimized. Fig. 3a shows the current-volt measurement of the DNA probe attachment on IDE. KOH-treated IDE shows the current level as 4.85 E−07 A, after adding amine-modified zeolite-IO, the current was increased to 4.15 E−06 A. This increment confirms the modification of zeolite-IO on IDE. Further, upon adding 200 nM of COOH-ended DNA probe, the current was further increased to 6.18 E−06 A, which confirms the interaction of DNA probe and APTES. Further, increasing the DNA probe concentrations to 400, 600, 800, and 1000 nM, the current responses also increased to 1.19 E−05 A, 1.92 E−05 A, 2.62 E−05 A, and 2.69 E−05 A, respectively. DNA probe with 600 and 800 nM shows approximately the similar current response indicating the saturation of DNA probe immobilization on IDE (Fig. 3b). During the initial stages of duplex formation, a twofold increase in current responses was observed, which then saturated with higher concentrations of the target DNA. From this experiment, an optimized concentration of 800 nM of DNA probe was desired to detect HPV-16 target DNA on the dual-probe station, supplying a current within the range of 0–2 V.

### 3.3 Detection of HPV-16 target DNA

**Fig. 4:**
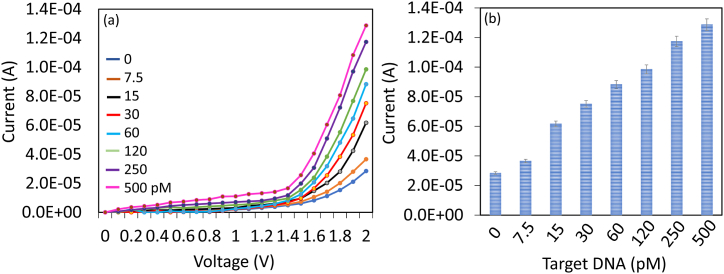
Detection of HPV-16 target DNA. (a) Current-volt measurement on hybridization with different concentrations of target DNA to the DNA probe. A clear increment in current was noticed with each target DNA. (b) The current level of hybridization of different concentrations of target DNA with the constant DNA probe. With increasing target DNA concentrations, current levels were gradually increased. Independent experiments were performed and averaged.

**Fig. 5:**
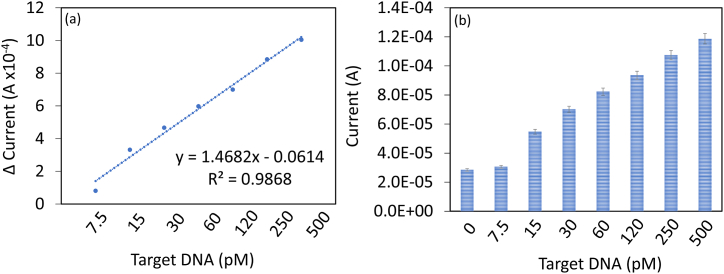
(a) Detection limit of target DNA. The difference in current response was plotted and the detection limit of the target to 7.5 pM at the R^2^ value of 0.9868. (b) Target DNA detection in spiked serum samples. Target DNA was spiked in human serum and added to DNA probe, and the current volt measurement was recorded. The current response was increased by enhancing the target DNA concentration, indicating the selective detection of target DNA. Independent experiments were performed and averaged.

HPV-16 target DNA was identified by capturing DNA on zeolite-IO immobilized IDE. Target DNA concentrations from 7.5 to 500 pM were dropped on DNA probe immobilized surface and then the current-volt measurements were recorded. Fig. 4a shows the graph of current-volt measurement of hybridization of different concentrations of target DNA with the immobilized DNA probe. Before adding the target DNA, the excess amine surface was blocked by PEG-COOH. PEG-COOH modified electrode shows the current response as 2.85 E−05 A, there is not much current response recorded after adding PEG-COOH due to the occupancy of DNA probe on the amine modified surfaces. When 7.5 pM of target DNA was added, the current response increased to 3.66 E−05 A, which confirms the hybridization of target DNA with DNA probe (Fig. 4a). Increasing the target DNA concentrations to 15, 30, 60, 120, 250, and 500 pM, current responses were gradually increased to 6.17 E−05 A, 7.52E-05 A, 8.83 E−05 A, 9.85 E−05 A, 1.17 E−04 A, and 1.29 E−04 A, respectively (Fig. 4b). The differences in current were calculated and plotted in an Excel sheet and calculated the detection limit of the target to 7.5 pM with an R^2^ value of 0.9868 (Fig. 5a). Based on the observed changes in current and the signal-to-noise ratio (3 sigma), it was evident that perfect duplex formation occurred at a lower picomolar level. This finding is consistent with the typical behavior observed in DNA sensors.

### 3.4 Identification of serum spiked target DNA

In any biosensor, identification of the target in the real-life sample is mandatory for diagnosing diseases. The biological sample contains an abundance of other proteins namely globulin, which interfere with the target identification and give the false-positive or false-negative result. Those proteins bind non-specifically to the sensing surface and lead to the false-positive. To reduce the biofouling, we used PEG-COOH as a blocking material to cover the excess amine surface. PEG-based molecules are efficiently used in various surface functionalization to reduce biofouling and enhance analytical performance. To confirm the selective detection of target DNA, it was spiked in diluted human serum and dropped on DNA probe modified, the current volt measurement was recorded. Fig. 5b shows the current response of serum-spiked target DNA binding with the DNA probe. As shown in the figure, the current response was increased by increasing the concentration of target DNA, indicating the selective detection of target DNA without interference from serum samples. In comparison to the target-spiked buffer, the target-spiked serum exhibited similar performance, despite the presence of several cross-molecular interferences in serum. Serum typically contains high levels of albumin and globulin, which are considered potential interfering molecules. Nevertheless, the current study performed well in the presence of serum under low current supply conditions.

### 3.5 Specificity and reproducibility of DNA biosensor on zeolite-IO modified IDE

**Fig. 6:**
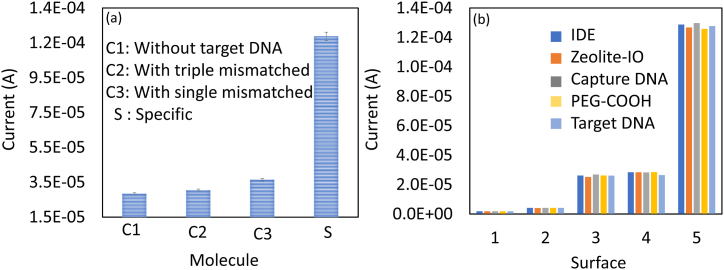
(a) Specific HPV-16 target DNA detection. Control performances with mismatched sequences were conducted. Single and triple mismatched sequences did not increase the current responses significantly, indicating the specific detection of HPV-16 target DNA. Independent experiments were performed and averaged. (b) Stability of the surface functionalization on interdigitated electrode. The surface modification and the target identification were done with five different sensors, all five sensor chips did not show any significant differences in current responses, which confirms the reproducibility of the DNA biosensor on zeolite-IO modified interdigitated electrode.

To confirm the specific detection of HPV-16 target DNA, control performances with single- and triple-mismatched sequences were conducted. As shown in Fig. 6a, single- and triple-mismatched sequences did not increase the current responses significantly. At the same time, target DNA increases the current responses due to the hybridization with DNA probe, indicating the specific detection of HPV-16 target DNA. Further, to check the stability of the surface functionalization on IDE, the same surface modification and the target identification was done with five different sensor chips, and the current-volt measurements were recorded. As shown in Fig. 6b, all five sensor chips did not show any significant differences in current responses, which confirms the reproducibility of the DNA biosensor on zeolite-IO modified IDE. The current responses between the specific (non-fouling) and non-specific (biofouling) interactions showed approximately a 6-fold difference, indicating excellent performance of the modified sensing surface. Overall, the current research introduces a highly sensitive detection method for the DNA sequence as a biomarker for HPV-16. This DNA biosensor utilizes a zeolite-IO modified IDE, which provides an expanded surface area due to the nanocomplex employed. By increasing the number of DNA probes, the target can form duplexes in higher numbers, resulting in improved detection performance. Moreover, the dipole-moment mechanism operating on the sensing surface enhances ionic movement towards the dielectrode, thereby facilitating interactive analysis monitoring. When positive and negative charges within a molecule separate, a net electric dipole is formed. As DNA molecules form a duplex on the detecting surface, changes in the dipole moment of the surface molecule and/or the surrounding environment occur due to this interaction. These variations in dipole moment can be identified by measuring electrical characteristics such as current. The attachment of DNA molecules to the sensor surface modifies the local electrical environment, thereby providing the basis for the detection method.

## 4 Conclusion

Cervical cancer originates in the cervix and can spread to nearby areas. The incidence of cervical cancer is increasing annually, posing a significant challenge to the medical industry and creating economic burdens. Human papillomavirus (HPV) is the primary culprit behind cervical cancer, entering the body through sexual contact. Regular HPV screening is crucial for identifying HPV infections before they progress to cancer. This research developed an HPV-16 DNA biosensor using zeolite-iron oxide (zeolite-IO) on an interdigitated electrochemical (IDE) sensor. Zeolite-IO was derived from coal mine fly ash using the sol-gel method, yielding nanoparticles approximately 20 nm in size, as confirmed by FESEM and FETEM analyses. The presence of Si–Al, Fe, and O was verified by EDX analysis. The extracted zeolite-IO was then attached to the IDE using an amine linker, followed by immobilization of COOH-ended DNA probes. Target DNA was successfully identified at concentrations as low as 7.5 pM on these surfaces. Furthermore, serum-spiked target DNA increased current response without interference, while single- and triple-mismatched sequences showed no significant changes in current responses, indicating selective and specific detection of target DNA. This IDE sensor, modified with nanomaterials, enables the detection of HPV-16 DNA at lower levels, facilitating the monitoring of cervical cancer and its progression. Use this DNA sensor technology in clinical settings to detect HPV infections early and for routine screening. This can assist in identifying those who are susceptible to cervical cancer and in directing the development of suitable care and treatment plans. Further, this DNA sensor can also be used to track the effectiveness of HPV vaccinations and evaluate how long HPV infections last.

## CRediT authorship contribution statement

**Ling Li:** Writing – review & editing, Writing – original draft, Methodology, Investigation, Funding acquisition, Formal analysis, Data curation, Conceptualization. **Subash C.B. Gopinath:** Writing – review & editing, Visualization, Validation, Supervision, Software, Resources, Project administration, Funding acquisition, Data curation, Conceptualization. **Thangavel Lakshmipriya:** Writing – review & editing, Validation, Resources, Formal analysis, Data curation. **Sreeramanan Subramaniam:** Writing – review & editing, Validation, Resources. **Periasamy Anbu:** Writing – review & editing, Validation, Resources.

## Declaration of competing interest

The authors declare that they have no known competing financial interests or personal relationships that could have appeared to influence the work reported in this paper.
